# Supercritical Carbon Dioxide Extraction of Flavonoids from Pomelo (*Citrus grandis* (L.) Osbeck) Peel and Their Antioxidant Activity

**DOI:** 10.3390/ijms131013065

**Published:** 2012-10-12

**Authors:** Jin-Zhe He, Ping Shao, Jian-Hua Liu, Qiao-Mei Ru

**Affiliations:** College of Biological and Environmental Engineering, Zhejiang University of Technology, Hangzhou 310032, China; E-Mails: hejzgd@163.com (J.-Z.H.); pingshao325@zjut.edu.cn (P.S.); jhliu@zjut.edu.cn (J.-H.L.)

**Keywords:** pomelo (*Citrus grandis* (L.) Osbeck) peel, flavonoids, supercritical carbon dioxide (SC-CO_2_) extraction, response surface methodology, antioxidant activity

## Abstract

Supercritical carbon dioxide (SC-CO_2_) extraction of flavonoids from pomelo (*Citrus grandis* (L.) Osbeck) peel and their antioxidant activity were investigated. Box-Behnken design combined with response surface methodology was employed to maximize the extraction yield of flavonoids. Correlation analysis of the mathematical-regression model indicated that a quadratic polynomial model could be used to optimize the SC-CO_2_ extraction of flavonoids. The optimal conditions for obtaining the highest extraction yield of flavonoids from pomelo peel were a temperature of 80 °C, a pressure of 39 MPa and a static extraction time of 49 min in the presence of 85% ethanol as modifier. Under these conditions, the experimental yield was 2.37%, which matched positively with the value predicted by the model. Furthermore, flavonoids obtained by SC-CO_2_ extraction showed a higher scavenging activity on hydroxyl, 1,1-diphenyl-2-picrylhydrazyl (DPPH) and 2,2′-azino-bis(3-ethylbenzthiazoline-6-sulphonic acid) (ABTS) radicals than those obtained by conventional solvent extraction (CSE). Therefore, SC-CO_2_ extraction can be considered as a suitable technique for the obtainment of flavonoids from pomelo peel.

## 1. Introduction

Supercritical fluid extraction (SFE) has been applied extensively by food and medical industries in recent years, since it is an environment-friendly technology that represents an alternative to conventional extraction methods and offers several advantages over conventional solvent extraction (CSE) methods [1]. Supercritical carbon dioxide (SC-CO_2_) is the most commonly used solvent in supercritical fluid extraction. SC-CO_2_ solvent has highly desirable properties such as non-toxicity, non-flammability, non-explosiveness, low cost, readily availability and ease of removal from the extracted materials [2–5]. Moreover, CO_2_ has a low critical temperature (*T*_c_ = 31.1 °C) and technically convenient critical pressure (*P*_c_ = 73.8 bar), which would prevent degradation of thermally labile and reactive components during extraction [6,7].

Pomelo (*Citrus grandis* (L.) Osbeck), belongs to the family Rutaceae and is a plant native to southeastern Asia. Wang *et al*. [8] reported that flavonoids have a high distribution of pomelo peel, reaching a concentration of approximately 46.7 mg/g dry weight. However, pomelo peel is normally thrown away as waste after the enjoyment of this fruit. Although numerous work has been done on flavonoid extraction from citrus peels and their antioxidant effect [9–12], no researches on flavonoids extraction from pomelo peel by SC-CO_2_ extraction technique and their antioxidant property are available until now.

In this study, SC-CO_2_ extraction technique was used to extract flavonoids from the pomelo peel. The efficiency of SC-CO_2_ extraction technique is affected by numerous factors, particularly temperature, pressure, supercritical CO_2_ flux, presence of a modifier and extraction time, which can affect the supercritical fluid selectivity, yield and extraction rate [13]. In order to maximize extraction yield, response surface methodology was employed to optimize the key parameters of SC-CO_2_ extraction. Moreover, the efficiency of SC-CO_2_ extraction of flavonoids was compared with that of CSE. Furthermore, the antioxidant activity of purified flavonoids of pomelo peel was determined by means of scavenging abilities of hydroxyl, 2,2-diphenyl-1-picrylhydrazyl (DPPH) and 2,2′-azino-bis(3-ethylbenzthiazoline-6-sulphonic acid) (ABTS) radicals.

## 2. Results and Discussion

### 2.1. Optimization of SC-CO_2_ Extraction Parameters by Response Surface Methodology

#### 2.1.1. Model Fitting

The experimental design and corresponding response values are listed in Table 1. The mathematical model representing the extraction yield of flavonoids as a function of the independent variables within the region under investigation was expressed by the following equation:

(1) $$Y=2.14+0.098x_{1}+0.11x_{2}+0.10x_{3}+0.064x_{1}x_{2}+0.16x_{1}x_{3}-0.005x_{2}x_{3}+0.032x_{1}^{2}-0.16x_{2}^{2}-0.29x_{3}^{2}$$

Analysis of variance (ANOVA) for the response surface quadratic model was used to explore the linearity and quadratic effect of the treatment variables, their interactions and coefficients on the response of the extraction yield (Table 2). The significance of each coefficient was determined by *p*-values. As shown in Table 2, *p*-values of the monomial coefficients *x*_1_, *x*_2_ and *x*_3_ were all less than 0.01, indicating that temperature, pressure and static extraction time as the linear terms were highly significant. Moreover, *p*-values for the quadratic coefficients *x*_1_^2^ and *x*_3_^2^ were significant (*p* < 0.01 and *p* < 0.001, respectively), which suggested that the quadratic terms of temperature and static extraction time had a significant effect on extraction yield. Furthermore, the *p*-value of the interaction coefficient *x*_1_*x*_3_ was lower than 0.01, indicating that the pairwise interaction model of temperature and static extraction time was highly significant, and there were significant interactions with extraction yield. However, the other two interaction coefficients *x*_1_*x*_2_ and *x*_2_*x*_3_ were insignificant (*p* > 0.05), indicating that interactions between temperature and pressure or interactions between pressure and static extraction time did not have effect on the extraction yield.

Generally, exploration and optimization of a fitted response surface may produce poor or misleading results unless the model exhibits a good fit, which makes checking the adequacy of the model essential [14]. The model would be more significant if the absolute *F*-value rises and the *p*-value lowers. The *F*-value and *p*-value of model were 17.31 and 0.0005, respectively, attesting that the model fitness was significant. Meanwhile, *F*-value for the lack of fit was insignificant (*p* = 0.1048) thereby implying the validity of the model. The fit of the polynomial model can be checked by the coefficient (*R^2^*) of determination [15]. The closer the value of adjusted *R^2^* is to 1, the better fit the model has and the better it predicts the response [16]. The value of adjusted *R*^2^ (0.9570) for Equation 1 suggested that the fitness between the predicted and actual values are good, where the total variation of 95.7% was attributed to the independent variables and only about 4.3% of the total variation cannot be explained by the model. Finally, the normal probability plot and the residual plot verified the assumptions of randomness, normality and constant variances of the residuals. Therefore, the quadratic model was found to be adequate in describing the response surface for the flavonoid extraction from pomelo peel.

#### 2.1.2. Response Surface Analysis

**Figure 1:**
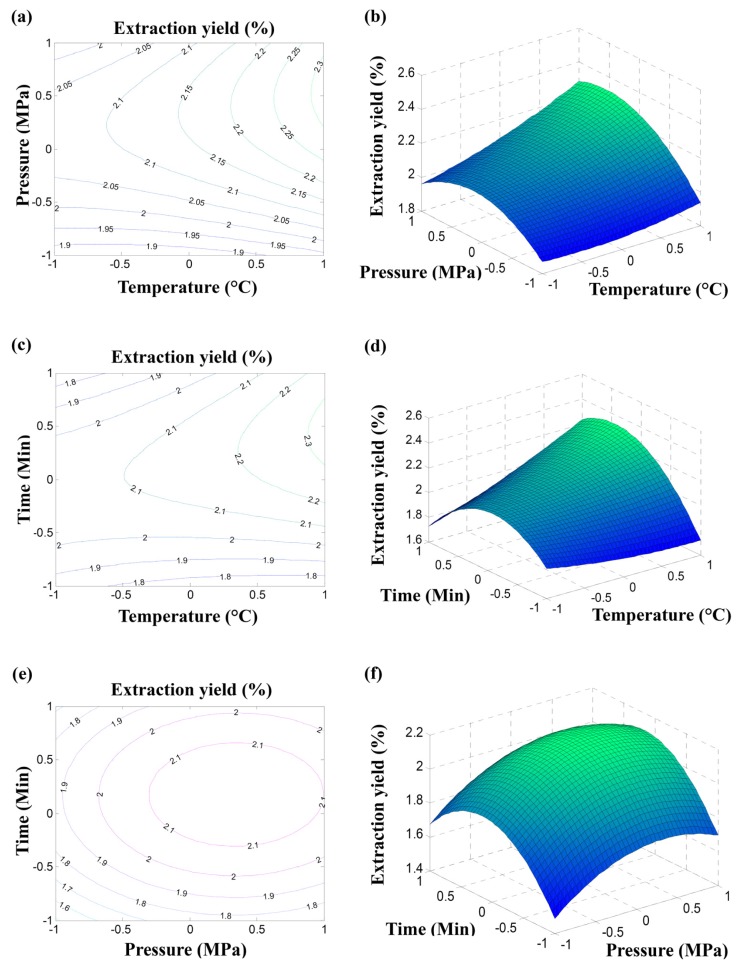
Contour (**a**, **c** and **e**) and 3-D response surface plots (**b**, **d**, and **f**) showing the effects of temperature, pressure and static extraction time on the extraction yield of flavonoids and their interactions. (**a**) and (**b**) at varying temperature and pressure, (**c**) and (**d**) at varying temperature and static extraction time, (**e**) and (**f**) at varying pressure and static extraction time.

The best way of expressing the effect of any independent variable on the flavonoid extraction yield is to generate surface response plots of the model, which were done by varying two variables within the experimental range under investigation and keeping the third variable at its “0” level [17]. The relationship between independent and dependent variables is illustrated in contour and 3-D response surface plots generated by the model for extraction yield (Figure 1). The response surface plots revealed that there was a region where neither an increasing nor a decreasing trend in the extraction capacity was observed, suggesting that there was an optimal condition for the extraction variables in order to maximize the extraction yield of flavonoids from pomelo peel.

The contour and 3-D response surface plots in Figure 1a,b shows the effects of pressure and temperature on the extraction yield of flavonoids at fixed static extraction time (level = 0, static extraction time = 40 min). By increasing pressure, the extraction yield was increased until reaching a highest value when the pressure was at 38–40 MPa. This could be due to the increase of solvent power and density of the SC-CO_2_ with the pressure [14]. However, further a increasing of the pressure resulted in little change in the extraction yield of flavonoids. The probable reason was that the highly compressed CO_2_ facilitates solute–solvent repulsion. Thus, high pressure is not always recommended, as it can potentially induce complex extraction and complicate the analysis [14]. When the temperature was increased from 60 to 80 °C, the extraction yield was increased from 1.94% to 2.35%. It was evident that temperature had a positive effect on extraction yield of flavonoids. Probably the increase of temperature could accelerate the mass transfer ratio, thus increasing the extraction yield [18]. This result suggested that operating at a higher temperature and a suitable pressure would favor the extraction of flavonoids from pomelo peel.

The contour and 3-D response surface plots of temperature and static extraction time at fixed pressure (level = 0, pressure = 35 MPa) are shown in Figure 1c,d. Similarly, increasing the temperature would increase the extraction yield of flavonoids and increasing the static extraction time, the extraction yield was also increased until an optimum value was reached at 45−52 min of static extraction time. However, further extending the reaction time resulted in little change in the extraction yield of flavonoids. These results were in accordance with previous studied [19,20]. By increasing the temperature from 60 to 80 °C, the extraction yield was increased from 1.97% to 2.34%. This indicated that operating at a higher temperature for a suitable static extraction time would promote the extraction of flavonoids from pomelo peel.

As shown in Figure 1e,f, extraction yield of flavonoids was affected by varying the pressure and static extraction time when the temperature was fixed at 70 °C (level = 0). It could be seen from the contour and 3-D response surface plots that the extraction yield was increased with increasing pressure until the pressure reached 39 MPa. In addition, the extraction yield of flavonoids was also increased until an optimum value was reached at 49 min of static extraction time. This indicated that operating at a suitable pressure and static extraction time would contribute to the extraction of flavonoids from pomelo peel. There was significant interaction between pressure and static extraction time. The static extraction time required to reach maximum yield was higher than that needed at higher-pressure levels.

#### 2.1.3. Optimum Conditions and Model Verification

The best fit extraction parameters can be validated by a stationary point of the optimized regression model. The stationary point is the combination of design variables where the surface is at either a maximum or a minimum in all directions, which can be found by using matrix algebra [21]. The solutions of stationary points of independent variables were calculated by using Design Expert software, and the results were: *x*_1_ = 0.981, *x*_2_ = 0.540 and *x*_3_ = 0.443.

According to Equation 2, the stationary points in terms of the natural variables were found:

(2) $$0.981=\frac{X_{1}-70}{10}\;\;\;0.540=\frac{X_{2}-35}{7}\;\;\;0.443=\frac{X_{3}-40}{20}$$

Thus, the calculated best fit extraction parameters were temperature of 79.81 °C, pressure of 38.78 MPa and static extraction time of 48.86 min, which were consistent with the optimal conditions of SC-CO_2_ extraction process to obtain the highest extraction yield selected on the basis of response surface (Figure 1).

The optimum extraction conditions (*X*_1_ = 79.81 °C, *X*_2_ = 38.78 MPa and *X*_3_ = 48.86 min) for the flavonoid extraction yield were estimated, and the predicted extraction yield of flavonoids from pomelo peel under the above conditions was 2.38%. To validate the predicted values, three additional validation runs were conducted. To ensure the predicted result was not biased toward the practical value, experiment rechecking was performed by using these modified optimal conditions: temperature of 80 °C, pressure of 39 MPa, and static extraction time of 49 min. The mean extraction yield for the flavonoids was 2.37% ± 0.083% (*n* = 3), corresponding well to the predicted value of the model equation, which confirmed that the response model was adequate for the optimization (Table 3).

### 2.2. Comparison of SC-CO_2_ Extraction and CSE

Different methods for natural matter extraction have different extraction yield and efficiency. A comparison of extraction yield of flavonoids between SC-CO_2_ extraction and CSE under optimal extracting conditions is shown in Table 4. Table 4 shows that SC-CO_2_ extraction could produce an extraction yield of 2.37% ± 0.083%, which was more efficient than CSE (1.51% ± 0.061%). In addition, the extraction time of SC-CO_2_ extraction was only 49 min, far lower than CSE (120 min). The results showed that SC-CO_2_ extraction was more efficient than CSE. Therefore, SC-CO_2_ extraction technique can be recommended as a suitable extraction method to isolate flavonoids from pomelo peel.

### 2.3. Antioxidant Property

**Figure 2:**
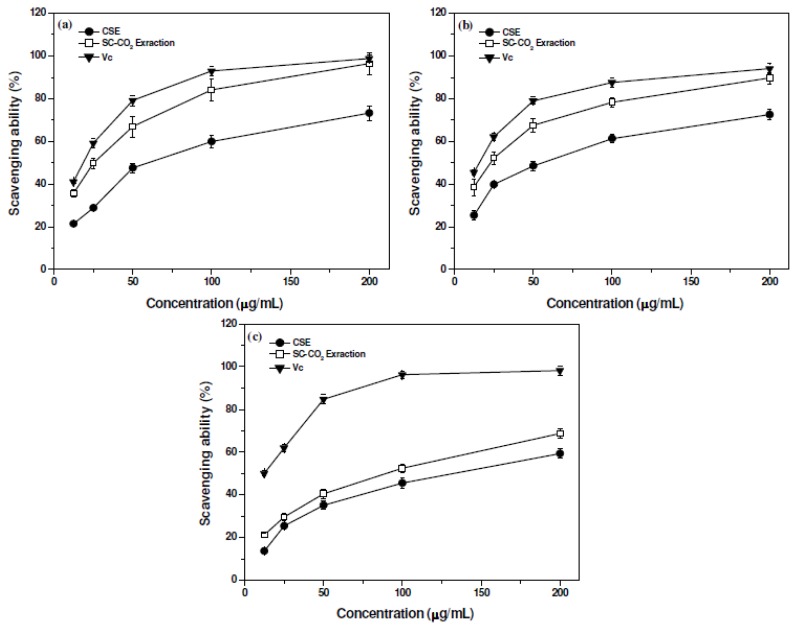
Scavenging abilities of the flavonoids extracted from pomelo peel on, DPPH (**a**), ABTS (**b**) and hydroxyl (**c**) radicals. Data are means ± standard deviation of triplicate experiments.

The model of scavenging the stable DPPH radicals has been widely accepted as a tool to evaluate the free radical-scavenging activities of materials [22]. Figure 2a describes the scavenging ability of purified flavonoids from pomelo peel on DPPH radicals. At all concentrations tested, flavonoids exhibited a dose-dependent DPPH radical-scavenging activity. The scavenging abilities of flavonoids extracted by SC-CO_2_ extraction and CSE on DPPH radicals were from 35.52% to 96.31% and from 21.43% to 73.25%, respectively, with concentration increasing from 12.5 to 200 μg/mL. The EC_50_ values of scavenging ability on DPPH radicals for flavonoids extracted by SC-CO_2_ extraction and CSE were 26 μg/mL and 60 μg/mL, respectively, indicating that flavonoids extracted by SC-CO_2_ extraction had a higher scavenging activity on DPPH radicals than that by CSE. The scavenging abilities of the flavonoids extracted by SC-CO_2_ extraction and CSE on DPPH radicals were all relatively lower than that of vitamin C (Vc) (EC_50_ = 22 μg/mL). However, at 200 μg/mL, the DPPH radical-scavenging activity of flavonoids extracted by SC-CO_2_ extraction was equivalent to the scavenging activity of Vc used in this study.

ABTS assay is often used in evaluating total antioxidant power of single compounds and complex mixtures of various plants [23]. Figure 2b reveals the scavenging ability of flavonoids from pomelo peel on ABTS radicals. In this assay, the concentration-dependent profile of scavenging activity on ABTS radicals was obvious for the tested flavonoids. The scavenging abilities of flavonoids extracted by SC-CO_2_ extraction and CSE on ABTS radicals were from 38.63% to 89.74% and from 25.35% to 75.23%, respectively, with concentration range of 12.5–200 μg/mL. Flavonoids extracted by SC-CO_2_ extraction revealed a better antioxidant activity because the EC_50_ values of scavenging ability on ABTS radicals for flavonoids extracted by SC-CO_2_ extraction and CSE were 23 μg/mL and 56 μg/mL, respectively. Although flavonoids of pomelo peel showed a lower scavenging activity on ABTS radicals than Vc (EC_50_ = 17 μg/mL), the ABTS radical-scavenging activity of flavonoids extracted by SC-CO_2_ extraction was to be equal to that of Vc at 200 μg/mL.

Removing hydroxyl radicals is important for the protection of living systems because they are considered to be mainly responsible for the oxidative injury of biomolecules [24]. Scavenging ability of the flavonoids isolated from pomelo peel on hydroxyl radicals is depicted in Figure 2c. In this work, the scavenging abilities of flavonoids extracted by SC-CO_2_ extraction and CSE on hydroxyl radicals were concentration-dependent, which were from 21.24% to 68.71% and from 13.72% to 59.33%, respectively, with concentration varying from 12.5 to 200 μg/mL. The EC_50_ values of scavenging ability on hydroxyl radicals for flavonoids extracted by SC-CO_2_ extraction and CSE were 80 μg/mL and 135 μg/mL, respectively, proving that flavonoids extracted by SC-CO_2_ extraction were better scavengers for hydroxyl radicals than those by CSE, although the scavenging ability of the flavonoids extracted by SC-CO_2_ extraction on hydroxyl radicals was relatively lower than that of Vc (EC_50_ = 12.5 μg/mL).

The antioxidant capacity of flavonoids from pomelo peel was assessed with the scavenging abilities of DPPH, ABTS and hydroxyl radicals. Our results indicated that flavonoids prepared by SC-CO_2_ extraction was found to have a significantly higher antioxidant activity than that obtained by CSE. The antioxidants are believed to donate hydrogen from the phenolic hydroxyl groups and break the free radical chain of oxidation forming a stable end product, which does not initiate or propagate further oxidation [25]. The high antioxidant activity of flavonoids can be attributed to hydroxy groups in the A- and B-rings, and the larger the number of hydroxy groups, the higher is the capacity to scavenge free radicals [26]. For flavonoids extracted by SC-CO_2_ extraction, their high antioxidant activity could be attributed to the high presence of phenolic hydroxyl group flavonoids.

## 3. Experimental Section

### 3.1. Plant Material and Chemicals

Pomelo was purchased from Yuhuan County, Zhejiang Province, China. The peel was washed and dried in an oven at 60 °C for 48 h to a moisture content of 8.0%, then ground to homogeneous powder (0.5 mm diameter) by an electrical food grinder.

Carbon dioxide (99.99% purity) supplied in a cylinder with a dip tube was purchased from Hangzhou Jinggong Specialty Gases Co., Ltd. (Hangzhou, China). ABTS, DPPH and Vitamin C (Vc) were purchased from Sigma Chemical Co. (St. Louis, MO, USA). All other chemicals were analytical grade and purchased from Shanghai Boer Chemical Reagent Co., Ltd. (Shanghai, China).

### 3.2. Supercritical CO_2_ Extraction

A supercritical fluid extractor Spe-ed SFE-2 (Applied Separation, USA) was used, which operates with two pumps, a master pump fitted with a cooling jacket on the pump head and a second pump (Knauer pump, model K-501, Berlin, Germany) for the addition of organic modifier. For extraction using SFE, 4 g dried pomelo peel powder was placed in a 30 mL extraction vessel. 85% aqueous ethanol was chosen as a modifier in this study. The static extraction was started when the desired pressure and specified temperature were reached. The operating pressure was provided by an air compressor. The extracted analyte was collected in glass vial with a rubber plug at the top. The CO_2_ flow rate was kept at approximately 1.0 mL/min by adjusting the outlet valve manually. The extraction was then performed under various experimental conditions in accordance with the experimental design. In order to assure the accuracy of the experimental data, the experiment was performed in triplicate.

### 3.3. Experimental Design

The three independent variables were *X*_1_, *X*_2_ and *X*_3_ representing temperature, pressure and static extraction time, respectively, while the dependent variable was the extraction yield. For statistical calculation, the variables were coded according to:

(3) $$x_{i}=\frac{X_{i}-X_{0}}{\delta X}$$

where *x_i_* is the coded value of an independent variable, *X_i_* is the real value of the independent variable, *X_0_* is the real value of an independent variable at the center point, and *δX* is the step change value.

The experimental runs were designed in accordance with a Box-Behnken design with three factors and three levels. According to the single factor experiments, the settings for independent variables were as follows for the low and high values: temperatures of 60 and 80 °C; pressures of 28 and 42 MPa; and static extraction time of 20 and 60 min. Each variable was coded at three levels: −1, 0 and +1. The symbols and levels are shown in Table 5. From the Box-Behnken design, seventeen experimental runs were required, and five replicates at the center (0, 0, 0) of the design were performed to allow the estimation of the pure error sum of squares.

Experimental data were fitted to a quadratic polynomial model using regression coefficients. The generalized quadratic polynomial model used in the response surface analysis was described in Equation 4:

(4) $$Y_{i}=\beta_{0}+\sum_{i=1}^{k}\beta_{i}x_{i}+\sum_{i=1}^{k}\beta_{ii}x_{i}^{2}+\sum \sum_{i\leq j}\beta_{ij}x_{ij}+{ɛ}_{i}$$

where *Y_i_* is the predicted response, *β_0_*, *β_i_*, *β_ii_* and *β_ij_* are the regression coefficients for intercept, linearity, square and interaction, respectively, while *x_i_* and *x_j_* are the independent code variables. The cross terms represent two-parameter interactions, and the quadratic terms represent second-order non-linearity, while ɛ is the statistical error that represents other sources of variability, such as measurement error.

### 3.4. Conventional Solvent Exraction (CSE)

Ten grams dried pomelo peel powder was placed in a reflux apparatus. Extraction was performed with 150 mL of 85% aqueous ethanol for 120 min at 85 °C. The crude extract was filtered. The solution was concentrated under reduced pressure. The procedure was performed in triplicate.

### 3.5. Determination of Flavonoid Content

The content of flavonoids was determined using a method described by Yi *et al*. [12] with several modifications. Briefly, 1 mL diluted sample was mixed with 1 mL of 5% (*w*/*w*) NaNO_2_. After 6 min, 1 mL of 10% (*w*/*w*) AlCl_3_ was added and allowed to stand for 6 min, then 5 mL of 4% (*w*/*w*) NaOH was added to the mixture. Absorbance was taken at 510 nm after 15 min. The content of flavonoids was expressed as rutin equivalents through the calibration curve of rutin. The calibration curve (*y* = 0.5923*x* − 0.0021, where *y* is absorbance of sample, *x* is sample concentration) ranged 10–1000 μg/mL (*R*^2^ = 0.9996).

### 3.6. Purification of Flavonoids by Macroporous Resin Adsorption

The crude flavonoids-enriched extract obtained under the optimized condition was purified using a column (25 × 1.5 cm^2^) packed with AB-8 macroporous adsorption resin according to the reference [17]. The conditions for purifying the flavonoids by AB-8 resin were: injecting concentration 3.75 mg/mL, pH = 5, injecting velocity 2.0 mL/min, 40% (*v*/*v*) ethanol as desorption solvent, desorption velocity of flow 1.5 mL/min. The purified extract of flavonoids was collected and evaporated at 50 °C, and was then freeze-dried for determination of antioxidant property.

### 3.7. Assay of Antioxidant Property

In order to evaluate antioxidant activity of flavonoids from pomelo peel, AB-8 macroporous adsorption resin was employed to purify the flavonoids obtained by SC-CO_2_ extraction and CSE. The antioxidant activity was investigated using biochemical methods of DPPH, ABTS and hydroxyl radical scavenging assay. Tests were carried out in triplicate.

The DPPH radical-scavenging activity was measured according to the method of Braca *et al*. [27]. Flavonoid sample with different concentrations (12.5–200 μg/mL, 1.0 mL) was mixed with methanol solution (3.0 mL) containing DPPH radicals (0.2 mM). The mixture was shaken vigorously and incubated for 30 min in darkness at room temperature, and then absorbance at 517 nm was measured.

The scavenging activity of the flavonoids against ABTS radicals (ABTS^+^) was measured using the method of Fellegrini *et al*. [28] with some modifications. ABTS^+^ were produced by reacting ABTS solution (7 mM, 25 mL) with potassium persulphate (1.4 mM, 0.44 mL), and the mixture was kept in the dark at room temperature for 12~16 h. In the moment of use, the ABTS^+^ solution was diluted with ethanol (475 mL) to an absorbance of 0.70 ± 0.02 at 734 nm. Flavonoid sample with different concentrations (12.5–200 μg/mL, 1.0 mL) was added to ABTS^+^ solution (3.0 mL) and mixed vigorously. After reaction at room temperature for 6 min, the absorbance at 734 nm was measured.

Hydroxyl radical-scavenging activity was determined based on the method described by Smirnoff and Cumbes [29] with some modifications. The reaction mixture contained flavonoid sample with different concentrations (12.5–200 μg/mL, 1 mL) was incubated with a solution containing orthophenanthroline (5 mM, 1 mL), phosphate buffer (7.5 mM, pH 7.4, 0.8 mL) and FeSO_4_ (7.5 mM, 0.5 mL). Finally, H_2_O_2_ (8.8 mM, 0.5 mL) was added, and the reaction mixture was then incubated at 37 °C for 1 h. The absorbance of the resulting solution was measured spectrophotometrically at 532 nm.

The radical scavenging ability was calculated using the following formula: scavenging ability (%) = (1 − *A*_sample_/*A*_control_) × 100, where *A*_control_ is the absorbance of control without the flavonoid sample, and *A*_sample_ is the absorbance in the presence of the flavonoid sample. The EC_50_ value (μg/mL) is the effective concentration at which the hydroxyl, DPPH or ABTS radicals are scavenged by 50%. Vc was used as reference compound.

### 3.8. Statistical Analysis

Each experiment was performed three times and the results were expressed as mean ± standard deviation of three replications. The software Design Expert (Trial Version 7.1.3.; Stat-Ease Inc.: Minneapolis, MN, USA) was used for experimental design, data analysis, quadratic model buildings, and graph (three-dimensional response surface and contour) plotting. *p* value < 0.05 was regarded as significant and *p* value < 0.001 as highly significant.

## 4. Conclusions

In this study, the SC-CO_2_ extraction of flavonoids from pomelo peel was investigated with three-variable (temperature, pressure and static extraction time), three-level experiment. Box-Behnken design and response surface methodology were applied to maximize the extraction yield of flavonoids. The optimal extraction conditions for the flavonoids were determined as follows: temperature of 80 °C, a pressure of 39 MPa, a static extraction time of 49 min and with 85% aqueous ethanol as modifier. Under these conditions, the experimental yield of flavonoids was 2.37% ± 0.083%, which was closed with the predicted yield value. Compared to CSE, SC-CO_2_ extraction had a higher extraction yield of flavonoids with lower extraction time. Furthermore, flavonoids obtained by SC-CO_2_ extraction showed better scavenging activities on hydroxyl, DPPH and ABTS radicals. Therefore, SC-CO_2_ extraction can be considered as an alternative to CSE for the obtainment of flavonoids from pomelo peel.

## Figures and Tables

**Table 1:** Box-Behnken design and response for the extraction yield of pomelo peel.

	Coded level			Response Extraction yield (%) a	
	
Run	*x*_1_ Temperature (°C)	*x*_2_ Pressure (MPa)	*x*_3_ Time (min)	Predicted	Experimental b
1	−1 (60)	−1 (28)	0 (40)	1.86	1.80 ± 0.081
2	1 (80)	−1 (28)	0 (40)	1.93	1.97 ± 0.093
3	−1 (60)	1 (42)	0 (40)	1.96	1.92 ± 0.027
4	1 (80)	1 (42)	0 (40)	2.29	2.35 ± 0.106
5	−1 (60)	0 (35)	−1 (20)	1.84	1.92 ± 0.065
6	1 (80)	0 (35)	−1 (20)	1.71	1.69 ± 0.046
7	−1 (60)	0 (35)	1(60)	1.72	1.74 ± 0.059
8	1 (80)	0 (35)	1 (60)	2.24	2.16 ± 0.110
9	0 (70)	−1 (28)	−1 (20)	1.48	1.46 ± 0.052
10	0 (70)	1 (42)	−1 (20)	1.69	1.65 ± 0.074
11	0 (70)	−1 (28)	1 (60)	1.67	1.71 ± 0.042
12	0 (70)	1 (42)	1 (60)	1.91	1.93 ± 0.063
13	0 (70)	0 (35)	0 (40)	2.14	2.19 ± 0.114
14	0 (70)	0 (35)	0 (40)	2.14	2.13 ± 0.092
15	0 (70)	0 (35)	0 (40)	2.14	2.07 ± 0.088
16	0 (70)	0 (35)	0 (40)	2.14	2.11 ± 0.056
17	0 (70)	0 (35)	0 (40)	2.14	2.18 ± 0.079

a Extraction yield is the percentage of the extracted flavonoids with respect to the dry weight of pomelo peel;

b Data are means ± standard deviation of triplicate experiments.

**Table 2:** Analysis of variance (ANOVA) and estimated regression coefficients for response surface quadratic model.

Source	Sum of squares	DF a	Mean square	*F*-Value	Prob > *F*
Linear					
*x*_1_	0.077	1	0.077	13.73	0.0076
*x*_2_	0.10	1	0.10	18.16	0.0037
*x*_3_	0.085	1	0.085	15.05	0.0061
Quadratic					
*x*_1_^2^	4.265 × 10^−3^	1	4.265 × 10^−3^	0.76	0.1308
*x*_2_^2^	0.11	1	0.11	19.16	0.0032
*x*_3_^2^	0.36	1	0.36	63.63	<0.0001
Interaction					
*x*_1_*x*_2_	0.017	1	0.017	2.93	0.1308
*x*_1_*x*_3_	0. 11	1	0.11	19.19	0.0032
*x*_2_*x*_3_	1.000 × 10^−3^	1	1.000 × 10^−4^	0.018	0.8978
Model	0.88	9	0.098	17.31	0.0005
Residual	0.039	7	5.639 × 10^−3^		
Lack of fit	0.030	3	9.902 × 10^−3^	4.06	0.1048
Pure error	9.767 × 10^−3^	4	2.442 × 10^−3^		
Cor total	0.92	16			
*R*^2^ = 0.9776					
Adj. *R*^2^ = 0.9570					

a Degree of freedom.

**Table 3:** Predicted and experimental values at optimum conditions.

Extraction conditions	Temperature (°C)	Pressure (MPa)	Extraction time (min)	Extraction yield (%) a
Optimum conditions	79.81	38.78	48.86	2.38 (predicted)
Modified conditions	80	39	49	2.37 ± 0.083 b (experimental)

a Extraction yield is the percentage of the extracted flavonoids with respect to the dry weight of pomelo peel;

b Data are mean ± standard deviation of triplicate experiments.

**Table 4:** Comparison of SC-CO_2_ extraction and CSE of extraction yield of flavonoids from pomelo peel.

Extraction method	Temperature (°C)	Extraction time (min)	Extraction yield (%) a
SC-CO_2_ extraction	80	49	2.37 ± 0.083 *
CSE	85	120	1.51 ± 0.061

a Flavonoid yield is the percentage of the extracted flavonoids with respect to the dry weight of pomelo peel; data are means ± standard deviation of triplicate experiments.

* Significant difference was determined at *p* < 0.05.

**Table 5:** Independent variables and their levels in the Box-Behnken design.

Independent variable	Coded symbol	Variable level		

		−1	0	1
Temperature (°C)	*x*_1_	60	70	80
Pressure (MPa)	*x*_2_	28	35	42
Time (min)	*x*_3_	20	40	60
